# Largazole Inhibits Ocular Angiogenesis by Modulating the Expression of VEGFR2 and p21

**DOI:** 10.3390/md19080471

**Published:** 2021-08-22

**Authors:** Beiying Qiu, Alison Tan, Yu Zhi Tan, Qi-Yin Chen, Hendrik Luesch, Xiaomeng Wang

**Affiliations:** 1 Centre for Vision Research, Duke NUS Medical School, 8 College Road, Singapore 169857, Singapore; beiying.qiu@duke-nus.edu.sg (B.Q.); alison.tan@duke-nus.edu.sg (A.T.); 2 Singapore Eye Research Institute (SERI), The Academia, 20 College Road, Level 6 Discovery Tower, Singapore 169856, Singapore; 3 Lee Kong Chian School of Medicine, Nanyang Technological University, 11 Mandalay Road, Singapore 308232, Singapore; tanyuzhi@gmail.com; 4 Department of Medicinal Chemistry and Center for Natural Products, Drug Discovery and Development (CNPD3), University of Florida, 1345 Center Drive, Gainesville, FL 32610, USA; chenqyufl@ufl.edu; 5 Institute of Molecular and Cell Biology (IMCB), Agency for Science, Technology and Research (A*STAR), Proteos, 61 Biopolis Dr, Singapore 138673, Singapore

**Keywords:** ocular angiogenesis, AMD, PDR, Largazole, cyclodepsipeptide

## Abstract

Ocular angiogenic diseases, characterized by abnormal blood vessel formation in the eye, are the leading cause of blindness. Although Anti-VEGF therapy is the first-line treatment in the market, a substantial number of patients are refractory to it or may develop resistance over time. As uncontrolled proliferation of vascular endothelial cells is one of the characteristic features of pathological neovascularization, we aimed to investigate the role of the class I histone deacetylase (HDAC) inhibitor Largazole, a cyclodepsipeptide from a marine cyanobacterium, in ocular angiogenesis. Our study showed that Largazole strongly inhibits retinal vascular endothelial cell viability, proliferation, and the ability to form tube-like structures. Largazole strongly inhibits the vessel outgrowth from choroidal explants in choroid sprouting assay while it does not affect the quiescent choroidal vasculature. Largazole also inhibits vessel outgrowth from metatarsal bones in metatarsal sprouting assay without affecting pericytes coverage. We further demonstrated a cooperative effect between Largazole and an approved anti-VEGF drug, Alflibercept. Mechanistically, Largazole strongly inhibits the expression of VEGFR2 and leads to an increased expression of cell cycle inhibitor, p21. Taken together, our study provides compelling evidence on the anti-angiogenic role of Largazole that exerts its function through mediating different signaling pathways.

## 1. Introduction

Abnormal blood vessel formation in the eye is a characteristic feature of many blinding eye diseases, such as neovascular age-related macular degeneration (nAMD) and proliferative diabetic retinopathy (PDR) [1]. nAMD is caused by vascular malformations in the choroidal vasculature, which becomes hyperproliferative and penetrates the retinal pigmented epithelium into the subretinal space, especially in the macular region of the retina [2]. nAMD is the leading cause of vision loss in adults aged over 65 years [3]. The number of people with AMD worldwide is expected to be around 288 million by 2040 [4]. PDR, on the other hand, is caused by excessive neovascularization in the light-sensing neuroretina, which may invade the vitreous body to cause severe vision impairment and even blindness. PDR is the most common cause of vision impairment in working-age adults, which has devastating personal and socioeconomic consequences [5,6]. The incidence of PDR increases substantially in patients with prolonged diabetes [7]. The overall prevalence of PDR in type 2 diabetes patients is 6%, while the prevalence of PDR in DR patients is 17% [8].

Advances in understanding molecular mechanisms of neovascularization led to the identification of vascular endothelial growth factor (VEGF) [9]. Multiple inhibitors targeting VEGF have been developed over the last two decades and have been widely used to treat ocular angiogenic diseases. VEGF blockers are effective in suppressing vessel leakage and inhibiting new blood vessel formation [10,11,12]. However, repeated treatment is required to achieve optimal treatment outcomes [13]. Considering the important neuroprotective role of VEGF, there is significant concern regarding potential side effects associated with long-term anti-VEGF treatment [14]. Importantly, approximately 50% of patients are reported to be nonresponsive to anti-VEGF therapy [15], and from 15% to 20% of patients may develop resistance to anti-VEGF drugs over time [16], which is likely due to the compensatory activation of alternative angiogenic pathways in the presence of anti-VEGF inhibitors. There is an urgent need to develop alternative or complementary treatment strategies to current anti-VEGF therapeutics.

Marine organisms serve as an emerging source for novel bioactive compounds, and many possess attractive pharmacological properties, including anti-angiogenic, anti-inflammatory, and anti-oxidant activities [17]. So far, more than 25,700 compounds with unique chemical structures have been extracted from marine organisms, like molluscs, soft corals, fishes, and many microorganisms [18]. We recently described a marine natural product inspired inhibitor of cotranslational translocation, Apratoxin S4, as a potent anti-angiogenic compound in vitro and ex vivo and as an inhibitor of pathological ocular neovascularization in vivo [19]. The cyclodepsipeptide Largazole, isolated from a marine cyanobacterium, has been shown to have potent anti-proliferative properties in multiple cancer cell lines by inhibiting class I histone deacetylases (HDACs) [20,21,22,23,24]. Largazole is a prodrug that liberates the active species, Largazole thiol, upon protein-assisted hydrolysis and could inhibit tumor growth and induce apoptosis in the tumor [24]. This effect is probably mediated by the regulation of cell cycle components, antagonism of the AKT pathway, and inhibition of EGFR signaling [24]. During angiogenesis, quiescent endothelial cells (ECs) are activated and become highly proliferative [25]. Largazole was previously reported to inhibit multiple pro-angiogenic pathways, including VEGF and TGFβ signaling pathways, as a result of class I HDAC inhibition [26,27]. It was also shown to attenuate inflammatory corneal neovascularization by downregulating various pro-angiogenic and upregulating anti-angiogenic factors [26]. However, Largazole’s effect in neovascularization at the posterior compartment of the eye remains to be evaluated. Here we studied the anti-angiogenic effect of Largazole in multiple in vitro and ex vivo models and dissected its mechanism of action.

## 2. Results

### 2.1. Largazole Inhibits the Activation of Human Retinal Endothelial Cells (HRECs) in a Dose-Dependent Manner

**Figure 1:**
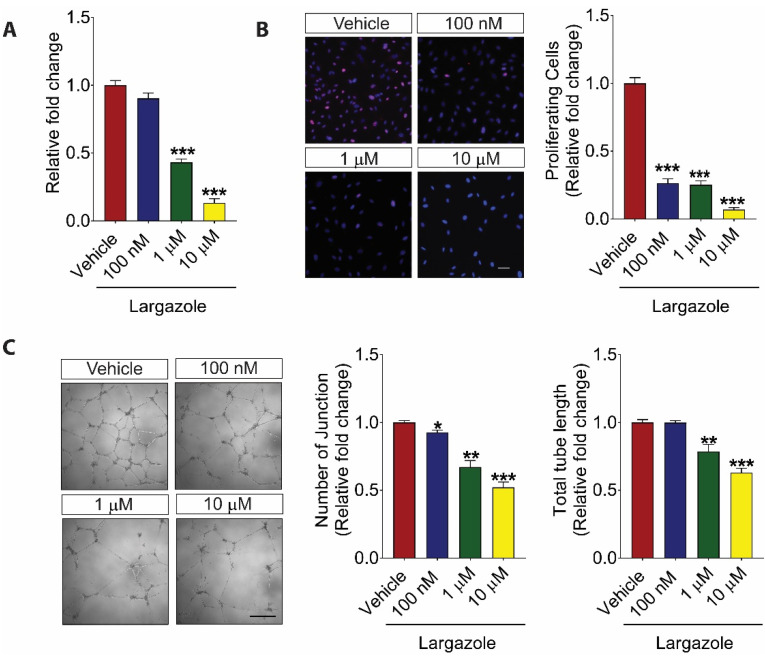
Largazole inhibits the activation of human retinal endothelial cells (HRECs) in a dose-dependent manner. (**A**) AlamarBlue assay demonstrated dose-dependent inhibition of HRECs proliferation by Largazole following 24 h treatment (n = 3). (**B**) Immunofluorescence staining (left) with Ki67 (red) and DAPI (blue) and quantitative analysis (right) demonstrating a dose-dependent inhibition of HREC proliferation by Largazole following 24 h treatment (n = 3). Scale bar: 50 μm. (**C**) Representative images (left) and quantification (right) of Largazole’s effect on HREC tube formation including total tube length and number of junctions in Matrigel following 16 h treatment (n = 3). Scale bar: 500 μm. All images shown are representative, and data are presented as means ± s.e.m. Statistical significance was determined by one-way ANOVA followed by Tukey’s multiple comparison test or unpaired, two-tailed Student’s *t*-test; * *p* < 0.05, ** *p* < 0.01, and *** *p* < 0.001.

As angiogenesis is a highly context-dependent process [28], we investigated the impact of Largazole on HREC activation. Our results showed that Largazole inhibited HREC viability in a dose-dependent manner after 24 h treatment (Figure 1A). The relative fold changes in cell viability following Largazole treatment were 0.90 ± 0.02 (100 nM), 0.43 ± 0.01 (1 μM), and 0.13 ± 0.02 (10 μM). To further understand the effect of Largazole on HREC proliferation, vehicle- and Largazole-treated HRECs were subjected to immunofluorescence staining with an antibody specific to Ki67, a cell proliferation marker. DAPI staining was used to label all cells following the treatment. Cell proliferation was determined by the percentage of Ki67 positive cells over DAPI positive cells. Consistent with the observation using Alamar Blue, Largazole significantly suppressed the HREC proliferation (Figure 1B). The relative fold changes in the percentage of Ki67 positive cells upon different Largazole’s doses were 0.26 ± 0.02 (100 nM), 0.25 ± 0.02 (1 μM), and 0.07 ± 0.01 (10 μM). During angiogenesis, endothelial cells align to form a tube-like structure [29]. Matrigel tube formation assay is one of the most widely used assays to mimic this process in vitro. Our study showed that Largazole blocked the HREC tube formation in a dose-dependent manner (Figure 1C). Specifically, the number of junctions was reduced by 7.33% ± 1.1% (100 nM), 32.83% ± 3.17% (1 μM), and 47.83% ± 2.57% (10 μM); and the total tube length was reduced by 21.53% ± 3.53% (1 μM) and 36.94% ± 2.06% (10 μM). Taken together, these results showed a potent anti-angiogenic role of Largazole in various HREC-based in vitro assays.

### 2.2. Largazole Inhibits Angiogenesis in Ex Vivo Models

**Figure 2:**
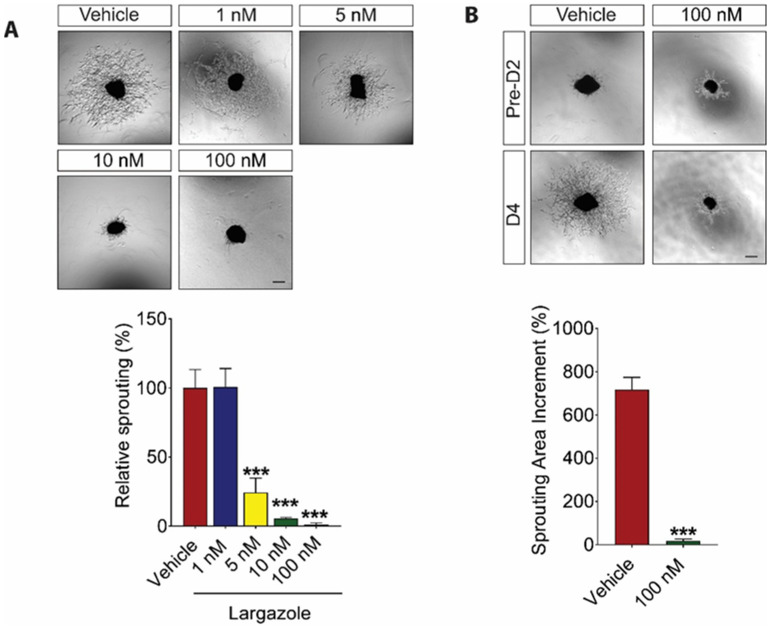
Largazole inhibits choroidal vessel outgrowth and has synergic manner with, while it has no impact on existing choroidal vessel. (**A**) Representative images (upper) and quantitative analysis (lower) of microvessel formation from mouse choroidal explants demonstrating a significant inhibitory effect of Largazole (n = 3 independent experimental groups, n ≥ 6 explants per treatment group). (**B**) Representative images (upper) and quantitative analysis (lower) of choroidal vessel outgrowth following Largazole treatment as compared to that before the treatment (n = 3 independent experimental groups, n ≥ 6 explants per treatment group). Pre-D2, images were taken after choroid explants have been embedded for 2 days. D4 images were taken after the choroid explants have been embedded for 4 days and treated with vehicle or Largazole for 2 days. Sprouting area increments were calculated used the following equation: (D4 vessel sprouting area / pre-D2 vessel sprouting area) × 100% − 100%. Scale bar: 100 μm. All images shown are representative, and data are presented as means ± s.e.m. Statistical significance was determined by one-way ANOVA followed by Tukey’s multiple comparison test or unpaired, two-tailed Student’s *t*-test; *** *p* < 0.001.

Abnormal choroid blood vessel formation contributes to the development of nAMD [30,31,32]. We first investigated Largazole’s effect on ocular-specific vessel growth using mouse choroid sprouting assay. Largazole demonstrated a dose-dependent inhibition on choroidal vessel outgrowth as compared to the vehicle-treated controls. The percentage of inhibition for 5 nM, 10 nM, and 100 nM Largazole was 75.68% ± 3.47%, 94.47% ± 1.11%, and 98.93% ± 0.41%, respectively (Figure 2A). Having established the role of Largazole in choroidal angiogenesis, we went on to study the effect of Largazole on fully established, quiescent choroidal vessels. Our study showed a 717.34% ± 56.88% increase in choroidal vessel outgrowth 4 days following the explant embedding (D4) as compared to that in day 2 following the explant embedding (D2) in the vehicle control group. On the contrary, 100 nM Largazole only led to around 17% increase in vessel outgrowth at D4 compared to that in D2 (Figure 2B). It is worth noting that 100 nM Largazole has no impact on the pre-existing choroidal vessel outgrowth.

**Figure 3:**
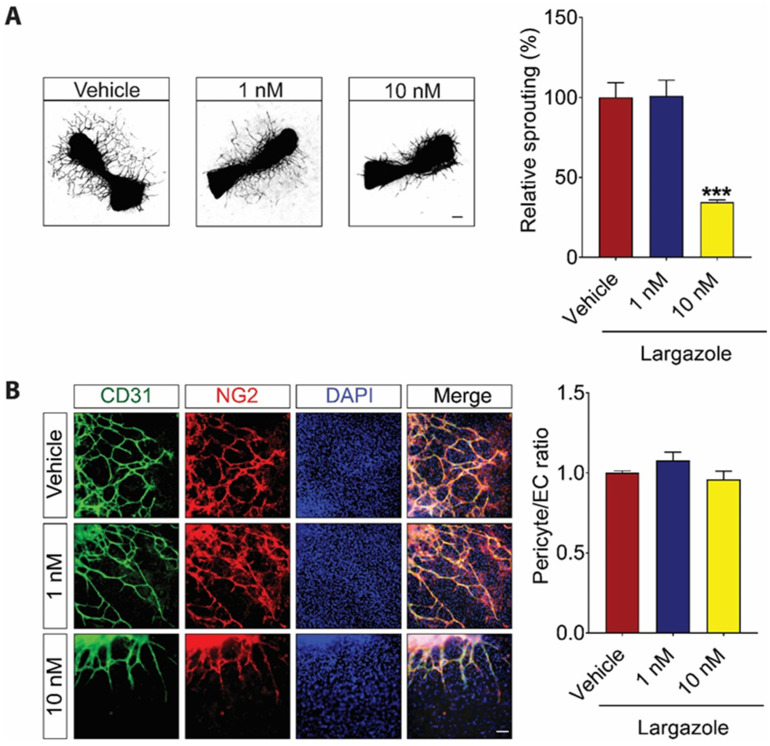
Largazole inhibits vessel outgrowth from metatarsal, while it has no impact on pericyte coverage. (**A**) Representative images (left) and quantitative analysis (right) of microvessel grown from mouse metatarsal explants demonstrating a significant anti-angiogenic effect of Largazole (n = 3 independent experimental groups, n ≥ 6 explants per treatment group). Scale bar: 100 μm. (**B**) Representative image (left) and quantitative analysis (right) of pericyte coverage of vessel outgrowth from mouse metatarsal explants demonstrating no significant changes in pericyte coverage following Largazole treatment (n = 3 independent experimental groups, n ≥ 6 explants per treatment group). CD31 (green), NG2 (red), and DAPI (blue) specifically stain endothelial cells, pericytes, and nucleus, respectively. Scale bar: 20 μm. All images shown are representative, and data are presented as means ± s.e.m. Statistical significance was determined by one-way ANOVA followed by Tukey’s multiple comparison test or unpaired, two-tailed Student’s *t*-test; *** *p* < 0.001.

Angiogenesis is a complex process that involves the interaction of multiple cell types, growth factors, and extracellular matrix components [28]. Mouse fetal metatarsal assay mimics the angiogenesis events in vivo in a multi-cellular environment [33]. We next investigated Largazole’s effect on pericyte coverage using the metatarsal assay. Following Largazole treatment, the blood vessel outgrowth from metatarsals was labeled using an endothelial cell marker, CD31. Similar to what was observed in vitro and choroidal assay, 10 nM but not 1 nM Largazole led to around 60% reduction in vessel outgrowth from metatarsal explants (Figure 3A). It is worth noting that the inhibitory effect of Largazole seems to be specific to ECs. No change in pericyte coverage was observed at the indicated concentrations (Figure 3B).

Taken together, these data showed a potent anti-angiogenic effect of Largazole in different multi-cellular ex vivo models. Largazole is able to prevent angiogenesis at nanomolar concentrations. Strikingly, the same dosage of Largazole does not affect pericyte coverage or pre-existing choroidal vasculature.

### 2.3. Largazole Showed Cooperative Effect to Aflibercept in Choroidal Angiogenesis Assays

**Figure 4:**
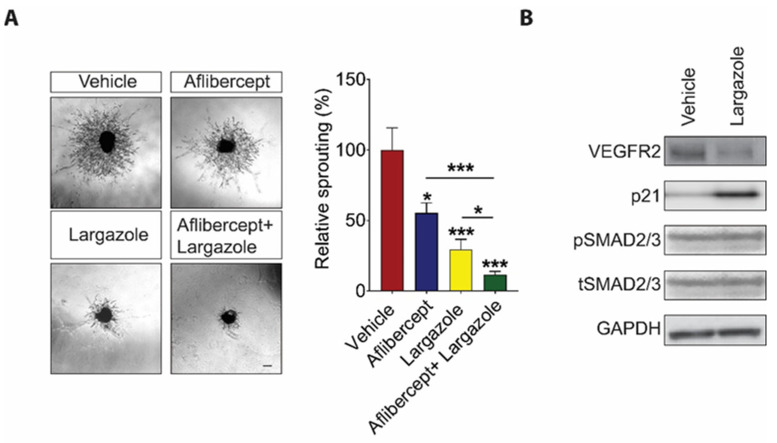
Largazole exerts anti-angiogenic effects through multiple signaling pathway. (**A**) 10 nM Largazole inhibits choroidal vessel outgrowth in cooperative manner with 500 µM Aflibercept. Representative images (left) and quantitative analysis (right) of microvessel formation from mouse choroidal explants (n = 3 independent experimental groups, n ≥ 6 explants per treatment group). Scale bar: 100 μm. All images shown are representative, and data are presented as means ± s.e.m. Statistical significance was determined by one-way ANOVA followed by Tukey’s multiple comparison test or unpaired, two-tailed Student’s *t*-test; * *p* < 0.05, and *** *p* < 0.001. (**B**) Largazole at concentration of 100 nM inhibits multiple angiogenic pathways. Representative western blots for VEGFR2, p21, pSMAD2/3, tSMAD2/3, and GAPDH in Largazole- and vehicle-treated HRECs (n = 3).

Anti-VEGF drugs are first-line treatments for a wide variety of ocular angiogenic diseases. However, a substantial number of patients are not responsive to the treatment. One possible mechanism behind the resistance to anti-VEGF therapies is the activation of alternative angiogenic pathways following anti-VEGF treatment [15,16,34]. There is thus an impetus to develop additional novel therapeutics to circumvent this problem. Having established the potent anti-angiogenic effect of Largazole, we went on to investigate whether Largazole works collaboratively with Aflibercept, an anti-VEGF molecule that is currently used in the clinic, in suppressing angiogenesis. Choroidal explants were treated with 5 nM Largazole and 500 µM Aflibercept either on their own or in combination. Although Aflibercept and Largazole inhibit choroidal angiogenesis by 33.60% ± 10.24% and 70.49% ± 5.28%, respectively, on their own, together, they led to a significantly stronger inhibition, which is 88.35% ± 1.81% on choroidal vessel outgrowth (Figure 4A).

### 2.4. Largazole Exerts Its Anti-Angiogenic Effects through Controlling VEGF Signaling and the Expression of Cell Cycle Inhibitor, p21

The impact of Largazole on cancer cell proliferation has been extensively studied [20,22,23,24,35,36]. To understand the mechanism of action for Largazole in ECs, Western blot analysis was performed on 100 nM Largazole treated HRECs. Consistent with the previous reports, Largazole significantly inhibits the expression of VEGFR2, which is a key receptor for VEGF in ECs during angiogenesis [37]. Furthermore, we found that the expression of p21, a cyclin-dependent kinase inhibitor, was upregulated drastically after treatment of Largazole for 24 h (Figure 4B). Although Largazole was reported to inhibit TGFβ signaling hepatic stellate cells [37], no difference was observed in pSmad2/3 phosphorylation in HRECs following Largazole treatment at concentration of 100 nM (Figure 4B). Taken together, our study showed that Largazole might exert an anti-angiogenic effect through suppressing the expression of VEGFR2 and inducing the expression of p21.

## 3. Discussion

In this study, we focused on investigating the anti-angiogenic role of Largazole in multiple ocular-specific in vitro and ex vivo pre-clinical models. Our results demonstrated a potent inhibitory effect of Largazole on HREC viability, proliferation, and the ability of HREC to a tube-like structure at 10 nM. We further showed that Largazole inhibits vessel outgrowth from choroid and metatarsal explants either on its own or in combination with Aflibercept, an anti-VEGF drug that is currently used in the clinic. Importantly, sprouting and angiogenic, instead of quiescent, blood vessels are more susceptible to Largazole. Mechanistically, Largazole exerts its function by suppressing the expression of a key VEGF receptor in endothelial cells, VEGFR2, and by inducing the expression of cell cycle inhibitor, p21.

Marine natural products constitute a tremendously large resource for novel active chemicals, which exhibit a wide variety of pharmacological properties. Marine bioactives have been explored for potential anti-angiogenic agents. As of 2013, more than 43 marine-derived active compounds show anti-angiogenic effects, and some of them have entered the different phases of clinical trials for cancer therapies [38]. Marine-derived compounds also show beneficial effects on combating angiogenesis in ocular angiogenic diseases. For example, Decapterus tabl ingredients could significantly inhibit retinal neovascular tufts by inhibiting HIF expression in the oxygen-induced retinopathy (OIR) mouse model [39]. Apratoxin S4, derived from a marine cyanobacterial compound, exhibits potent anti-angiogenic effects in OIR mouse models, a mouse model of laser-induced choroidal neovascularization, and persistent retinal neovascularization rabbit model through mediating multiple angiogenic pathways [19]. Largazole was identified as one of the marine cyanobacteria metabolites with a novel chemical structure from a cyanobacterium of the genus *Symploca* and later reclassified as *Caldora penicillata* [20,40,41]. Small molecules have been studied extensively in ocular angiogenesis [42,43]. However, the effective dosages are relatively high. Our study showed that Largazole is able to inhibit HREC activation and angiogenesis at nanomolar concentration. Largazole was previously reported to inhibit the proliferation of various types of cancer cells versus non-transformed cells [20,44]. In order to form new vascular tubes during angiogenesis, endothelial cells become highly proliferative [25]. Similar to what was observed in cancer cells, our study showed that Largazole is able to inhibit the viability and proliferation of activated HRECs as well as the vessel outgrowth from choroid and metatarsal explants at nanomolar concentration. One of the concerns with cyanobacterial and other natural products is non-specific cellular toxicity towards various cells [45]. In our study, the concentration of Largazole at 100 nM, which is 10 times more than the effective dosage of Largazole in various pre-clinical models of angiogenesis, does not affect the pre-existing choroidal vessels. Furthermore, despite being effective in inhibiting HREC activation and angiogenesis, 10 nM Largazole does not affect the pericyte coverage in the metatarsal assay. These observations suggest the possibility of using Largazole as a drug for ocular angiogenic diseases without causing substantial side effects and toxicity. However, a more thorough toxicity study needs to be carried out to understand the therapeutic index of Largazole, although current data are promising [23,24]. Single ip dosing with Largazole is up to 200 mg/kg, and repeated dosing on four consecutive days, using 60 mg/kg (×4) and 75 mg/kg (×4), did also not cause toxicity [23]. Although being effective in suppressing further angiogenesis and inhibiting blood vessel leakage [46,47], a substantial number of patients with PDR and nAMD are not fully responsive to the anti-VEGF treatment [48,49], and some may develop resistance over time [50], which is likely due to the compensatory activation of other angiogenic pathways in the presence of VEGF inhibitors. Combination treatment may provide an opportunity to solve such problems. Largazole was previously reported to inhibit the expression of VEGF, VEGF receptor, and its downstream signalling transducers in heptatic stellate cells and in ocular tissue of a mouse model of corneal angiogenesis [26,37]. However, our study does show a cooperative anti-angiogenic effect between Largazole and Aflibercept on choroidal vessel outgrowth, suggesting a non-VEGF dependent role of Largazole. Indeed, our results show that Largazole strongly induced the expression of cell cycle inhibitor p21.

In summary, our study demonstrated a potent anti-angiogenic effect of Largazole in various pre-clinical models of ocular angiogenesis and Largazole functions through modulating both VEGF-dependent and independent pathways. Further studies need to be carried out to understand the complete toxicity profile of Largazole, either on its own or in combination with Aflibercept. In addition, it will be interesting to explore Largazole’s mechanism of action mediated by histones or non-histone HDAC targets. Finally, in vitro and in vivo studies should be carried out to explore the possibility of using Largazole as an alternative treatment for patients who are less responsive to current anti-VEGF drugs.

## 4. Materials and Methods

### 4.1. Animals

Male and female C57BL/6J mice were purchased from Invivos (Singapore) and were kept on a 12 h light-dark cycle and fed a standard rodent chow (NCD, 18% kcal from fat, Harlan). The ex vivo assays, which require animals, were performed in compliance with the guidelines of the Institutional Animal Care and Use Committee of Singapore Eye Research Institute (SERI) (2020/SHS/1592) and the Association for Research in Vision and Ophthalmology Statement for the Use of Animals in Ophthalmic and Vision Research.

### 4.2. Materials, Cells, and Cell Culture

Primary human retinal endothelial cells (HRECs) were purchased from Angioproteomie (Boston, MA, USA) and maintained in endothelial growth media 2 (EGM2), which contained EBM2 basal media and Endothelial Cell Growth Medium BulletKits^TM^ (Lonza, Basel, Switzerland) according to the supplier’s instruction. Synthetic Largazole, generated similarly as previously described [21,51], was provided by Oceanyx Pharmaceuticals, Inc. (Woburn, MA, USA) and dissolved in DMSO.

### 4.3. AlamarBlue Cell Viability Assay

A total of 4 × 10^3^ HRECs were cultured in EGM2 media containing vehicle or Largazole for 24 h. AlamarBlue (Life Technology, Carlsbad, CA, USA) solution was incubated with HRECs for 4 h to allow viable cells to be labeled. The colorimetric signal was captured by Infinite M200 Pro (Tecan, Männedorf, Switzerland). The relative viability of HREC in the treatment group was determined by normalizing the absolute absorbance reading from the Largazole-treated group to that of vehicle-treated control.

### 4.4. Ki67 Proliferation Assay

A total of 8 × 10^3^ HRECs were cultured in EGM2 media containing vehicle or Largazole for 24 h. The cells were fixed in 4% PFA followed by 1 h incubation in blocking buffer (3% Triton X, 1% Tween 20, and 0.5% BSA in PBS) at room temperature. The HRECs were then incubated with Ki67 antibody (Abcam, Cambridge, UK) overnight at 4 °C followed by several times of washing with blocking buffer before being incubated with the secondary antibody and DAPI for 2 h at room temperature. Cells were visualized under the Zeiss AXIO (Zeiss, Oberkochen, Germany). Cell proliferation rate was determined by dividing the number of Ki67-positive cells by the total number of cells as labeled by DAPI, which was then normalized to that in vehicle-treated controls.

### 4.5. Matrigel Assay

A total of 1 × 10^4^ HRECs in Largazole or vehicle-containing EGM2 medium were seeded on top of growth factor reduced (GFR) Matrigel (BD Biosciences, Oxford, UK). Images were taken 16 h later under the Zeiss AXIO (Zeiss, Oberkochen, Germany). Total tube length and the number of junctions were analyzed using Image J Angiogenesis Analyzer (Version: 64-bit Java 1.8.0_112, Image J, Bethesda, MD, USA).

### 4.6. Choroid Sprouting Assay

Choroid sprouting assay was performed as described [32]. For the prevention study, treatment media containing either DMSO or Largazole at concentration of 1 nM, 5 nM, 10 nM, and 100 nM were added to the explants on the day of explant embedding. Media were changed every other day. Images were taken after 4 days of treatment. For regression assay, choroidal explants were allowed to grow for 2 days before being subjected to DMSO or 100 nM Largazole for 2 more days. For the additive study, choroid explants were treated with 500 µM Aflibercept, 5 nM Largazole, or a combination of both. Images were taken after 4 days of treatment. Vessel outgrowth was visualized under the Eclipse Ti-E Inverted Research Microscope (Nikon, Tokyo, Japan). The sprouting area was quantified by TRI2 software (Version: 3.0.1.2, TRI2, Oxyford, UK).

### 4.7. Metatarsal Assay

The metatarsal assay was performed as described [33]. In brief, metatarsal bones were isolated from E16.5 C57BL/6J mice and seeded onto gelatin-coated 24-well plates. Metatarsal explants were treated with DMSO or Largazole at concentration of 1 nM and 10 nM containing media 2 days after explant embedding, and media were changed every other day. On day 10 of culture, the explants were fixed in 4% PFA followed by 1 h incubation in blocking buffer (3% Triton X, 1% Tween 20, and 0.5% BSA in PBS). The explants were then incubated with CD31 antibody (553370, rat monoclonal, BD Biosciences, Franklin Lakes, NJ, USA) for staining ECs and NG2 antibody (AB5320, rabbit polyclonal, Millipore, Burlington, MA, USA) for staining pericytes overnight at 4 °C followed by few times of washing with blocking buffer before being incubated with the secondary antibodies for 2 h at room temperature. Vessel outgrowth from metatarsals was visualized under the Eclipse Ti-E Inverted Research Microscope (Nikon, Tokyo, Japan). Total vessel area was quantified using TRI2 software (Version: 3.0.1.2, TRI2, Oxyford, UK). NG2 intensity and CD31 intensity were measured AnigioTool software (Version: 0.6a (02.18.14), AngioTool64, Cambridge, UK). Pericyte coverage was determined by NG2 intensity divided by CD31 intensity.

### 4.8. Western Blotting

A total of 2 × 10^5^ HRECs were cultured in EGM2 media containing DMSO or 10 nM Largazole for 24 h Total protein was extracted using Tortex lysis buffer (20 mM HEPES, pH 7.9, 350 mM NaCl, 20% *v/v* glycerol, 1% NP-40, 1 mM MgCl_2_, 0.5 mM EDTA, 0.1 mM EGTA) and was separated by SDS-PAGE before being transferred onto a Immobilon-PSQ PVDF Membrane (#ISEQ-00010, Merck Millipore, Burlington, MA, USA). Blots were probed with VEGFR2 antibody (ab39256, rabbit polyclonal, Abcam, Cambridge, UK), p21 (2947, rabbit monoclonal, Cell signaling technology, Danvers, MA, USA), pSMAD2/3 antibody (8828, rabbit monoclonal, Cell signaling technology, Danvers, MA, USA), SMAD2/3 antibody (3102, Rabbit polyclonal, Cell signaling technology, Danvers, MA, USA), or glyceraldehyde 3-phosphate dehydrogenase (GAPDH) antibody (sc32233, mouse monoclonal, Santa Cruz Biotechnology, Dallas, TX, USA), followed by horseradish peroxidase (HRP)-conjugated secondary antibodies (Santa Cruz Biotechnology, Dallas, TX, USA).

### 4.9. Statistical Analysis

Data were presented as mean ± s.e.m. Statistical significance was performed using two-tailed, unpaired, Student’s *t*-test when we compared the treatment efficacy of 100 nM Largazole and vehicle control (Figure 2B), whereas one-way ANOVA followed by a Tukey post-test analysis was used for the rest of the studies where the efficacy of multiple treatment groups was compared using GraphPAD Prism (Version: 7.04, GraphPAD Software Inc., San Diego, CA, USA). * *p* < 0.05; ** *p* < 0.01; *** *p* < 0.001. Each represents significant statistical comparisons among the listed (x-axis) experimental groups.

## 5. Conclusions

In summary, our study provides compeling evidence for the anti-angiogenic effect of Largazole. Largazole inhibits EC viability, proliferation, and tube formation in a dose-dependent manner. It also inhibits metatarsal and choroidal vessel outgrowth. Importantly, Largazole, at the tested concentration, has no impact on pericyte coverage and pre-existing vessels. Finally, Largazole demonstrates a cooperative effect with Aflibercept on choroidal vessel outgrowth. Mechanistically, Largazole targets the VEGF signaling pathway and cell cycle mediator through class I HDAC inhibition.

## References

[1] Dreyfuss J.L., Giordano R.J., Regatieri C.V. (2015). Ocular Angiogenesis. J. Ophthalmol..

[2] Coleman H.R., Ferris F.L., Chan C.-C., Chew E.Y. (2008). Age-related macular degeneration. Lancet.

[3] Bressler N.M. (2004). Age-Related Macular Degeneration Is the Leading Cause of Blindness. JAMA.

[4] Wong W.L., Su X., Li X., Cheung C.M.G., Klein R., Cheng C.-Y., Wong T.Y. (2014). Global prevalence of age-related macular degeneration and disease burden projection for 2020 and 2040: A systematic review and meta-analysis. Lancet Glob. Health.

[5] Jampol L.M., Glassman A.R., Sun J. (2020). Evaluation and Care of Patients with Diabetic Retinopathy. N. Engl. J. Med..

[6] Wong T.Y., Tan B., Chua J., Lin E. (2016). Diabetic retinopathy. Nat. Rev. Dis. Prim..

[7] Fong D.S., Aiello L., Gardner T.W., King G.L., Blankenship G., Cavallerano J.D., Ferris F., Klein R. (2003). Retinopathy in Diabetes. Diabetes Care.

[8] Yang Q.-H., Zhang Y., Zhang X.-M., Li X.-R. (2019). Prevalence of diabetic retinopathy, proliferative diabetic retinopathy and non-proliferative diabetic retinopathy in Asian T2DM patients: A systematic review and Meta-analysis. Int. J. Ophthalmol..

[9] Hoeben A., Landuyt B., Highley M.S., Wildiers H., Van Oosterom A.T., De Bruijn E.A. (2004). Vascular Endothelial Growth Factor and Angiogenesis. Pharmacol. Rev..

[10] Writing Committee for the Diabetic Retinopathy Clinical Research (2015). Panretinal Photocoagulation vs Intravitreous Ranibizumab for Proliferative Diabetic Retinopathy: A Randomized Clinical Trial. JAMA.

[11] Ip M.S., Domalpally A., Hopkins J.J., Wong P., Ehrlich J.S. (2012). Long-term Effects of Ranibizumab on Diabetic Retinopathy Severity and Progression. Arch. Ophthalmol..

[12] Mitchell P., Liew G., Gopinath B., Wong T.Y. (2018). Age-related macular degeneration. Lancet.

[13] Wang W., Lo A.C.Y. (2018). Diabetic Retinopathy: Pathophysiology and Treatments. Int. J. Mol. Sci..

[14] Kurihara T., Westenskow P., Bravo S., Aguilar E., Friedlander M. (2012). Targeted deletion of Vegfa in adult mice induces vision loss. J. Clin. Investig..

[15] Diabetic Retinopathy Clinical Research (2015). Aflibercept, bevacizumab, or ranibizumab for diabetic macular edema. N. Engl. J. Med..

[16] Ciulla T., Hussain R.M., Ciulla L.M., Sink B., Harris A. (2016). Ranibizumab for diabetic macular edema refractory to multiple prior treatments. Retinoids.

[17] Lordan S., Ross R.P., Stanton C. (2011). Marine Bioactives as Functional Food Ingredients: Potential to Reduce the Incidence of Chronic Diseases. Mar. Drugs.

[18] Blunt J.W., Copp B.R., Keyzers R.A., Munroa M.H., Prinsepd M.R. (2016). Marine natural products. Nat. Prod. Rep..

[19] Qiu B., Tan A., Veluchamy A.B., Li Y., Murray H., Cheng W., Liu C., Busoy J.M., Chen Q.-Y., Sistla S. (2019). Apratoxin S4 Inspired by a Marine Natural Product, a New Treatment Option for Ocular Angiogenic Diseases. Investig. Opthalmology Vis. Sci..

[20] Taori K., Paul V.J., Luesch H. (2008). Structure and activity of largazole, a potent antiproliferative agent from the Floridian marine cyanobacterium Symploca sp.. J. Am. Chem. Soc..

[21] Ying Y., Taori K., Kim H., Hong J., Luesch H. (2008). Total Synthesis and Molecular Target of Largazole, a Histone Deacetylase Inhibitor. J. Am. Chem. Soc..

[22] Hong J., Luesch H. (2012). Largazole: From discovery to broad-spectrum therapy. Nat. Prod. Rep..

[23] Al-Awadhi F.H., Lilibeth A., Salvador-Reyes A., Lobna A.E., Ranjala R., Chen Q.-C., Luesch H. (2020). Largazole is a Brain-Penetrant Class I HDAC Inhibitor with Extended Applicability to Glioblastoma and CNS Diseases. ACS Chem. Neurosci..

[24] Liu Y., Salvador L.A., Byeon S., Ying Y., Kwan J.C., Law B.K., Hong J., Luesch H. (2010). Anticolon Cancer Activity of Largazole, a Marine-Derived Tunable Histone Deacetylase Inhibitor. J. Pharmacol. Exp. Ther..

[25] Ucuzian A.A., Gassman A.A., East A.T., Greisler H.P. (2010). Molecular Mediators of Angiogenesis. J. Burn. Care Res..

[26] Zhou H., Jiang S., Chen J., Ren X., Jin J., Su S.B. (2014). Largazole, an inhibitor of class I histone deacetylases, attenuates inflammatory corneal neovascularization. Eur. J. Pharmacol..

[27] Bousquet M.S., Ma J.J., Ratnayake R., Havre P.A., Yao J., Dang N.H., Paul V.J., Carney T.J., Dang L.H., Luesch H. (2016). Multidimensional Screening Platform for Simultaneously Targeting Oncogenic KRAS and Hypoxia-Inducible Factors Pathways in Colorectal Cancer. ACS Chem. Biol..

[28] Potente M., Gerhardt H., Carmeliet P. (2011). Basic and Therapeutic Aspects of Angiogenesis. Cell.

[29] Donovan D., Brown N., Bishop E., Lewis C. (2001). Comparison of three in vitro human angiogenesis assays with capillaries formed in vivo. Angiogenesis.

[30] Pemp B., Schmetterer L. (2008). Ocular blood flow in diabetes and age-related macular degeneration. Can. J. Ophthalmol..

[31] Hidayat A.A., Fine B.S. (1985). Diabetic choroidopathy. Light and electron microscopic observations of seven cases. Ophthalmology.

[32] Shao Z., Friedlander M., Hurst C.G., Cui Z., Pei D.T., Evans L.P., Juan A.M., Tahir H., Duhamel F., Chen J. (2013). Choroid sprouting assay: An ex vivo model of microvascular angiogenesis. PLoS ONE.

[33] Song W., Fhu C.W., Ang K.H., Liu C.H., Johari N.A.B., Lio D., Abraham S., Hong W., Moss S.E., Greenwood J. (2015). The fetal mouse metatarsal bone explant as a model of angiogenesis. Nat. Protoc..

[34] Cabral T., Mello L.G.M., Lima L.H., Polido J., Regatieri C.V., Mahajan V.B. (2017). Retinal and choroidal angiogenesis: A review of new targets. Int. J. Retin. Vitr..

[35] Zeng X., Yin B., Hu Z., Liao C., Liu J., Li S., Li Z., Nicklaus M.C., Zhou G., Jiang S. (2010). Total Synthesis and Biological Evaluation of Largazole and Derivatives with Promising Selectivity for Cancers Cells. Org. Lett..

[36] Law M.E., Corsino P.E., Jahn S.C., Davis B.J., Chen S., Patel B., Pham K., Lu J., Sheppard B., Nørgaard P. (2012). Glucocorticoids and histone deacetylase inhibitors cooperate to block the invasiveness of basal-like breast cancer cells through novel mechanisms. Oncogene.

[37] Liu Y., Wang J., Whang Z., Lam W., Kwong S., Li F., Friedman S.L., Zhou S., Ren Q., Xu Z. (2013). A histone deacetylase inhibitor, largazole, decreases liver fibrosis and angiogenesis by inhibiting transforming growth factor-beta and vascular endothelial growth factor signalling. Liver Int..

[38] Wang Y.-Q., Miao Z.-H. (2013). Marine-Derived Angiogenesis Inhibitors for Cancer Therapy. Mar. Drugs.

[39] Shoda C., Miwa Y., Nimura K., Okamoto K., Yamagami S., Tsubota K., Kurihara T. (2020). Hypoxia-Inducible Factor Inhibitors Derived from Marine Products Suppress a Murine Model of Neovascular Retinopathy. Nutrition.

[40] Salvador-Reyes L.A., Engene N., Paul V.J., Luesch H. (2015). Targeted Natural Products Discovery from Marine Cyanobacteria Using Combined Phylogenetic and Mass Spectrometric Evaluation. J. Nat. Prod..

[41] Engene N., Tronholm A., Salvator-Ryese L.A., Luesch H., Paul V.J. (2015). Caldora penicillata gen. nov., comb. nov. (cyanobacteria), a pantropical marine species with biomedical relevance. J. Phycol..

[42] Wojnarowicz P.M., E Silva R.L., Ohnaka M., Lee S.B., Chin Y., Kulukian A., Chang S.-H., Desai B., Escolano M.G., Shah R. (2019). A Small-Molecule Pan-Id Antagonist Inhibits Pathologic Ocular Neovascularization. Cell Rep..

[43] Sulaiman R.S., Merrigan S., Quigley J., Qi X., Lee B., Boulton M.E., Kennedy B., Seo S.-Y., Corson T.W. (2016). A novel small molecule ameliorates ocular neovascularisation and synergises with anti-VEGF therapy. Sci. Rep..

[44] Ying Y., Liu Y., Byeon S.R., Kim H., Luesch H., Hong J. (2008). Synthesis and Activity of Largazole Analogues with Linker and Macrocycle Modification. Org. Lett..

[45] DeMay J., Bernard C., Reinhardt A., Marie B. (2019). Natural Products from Cyanobacteria: Focus on Beneficial Activities. Mar. Drugs.

[46] Rosenfeld P.J., Brown D.M., Heier J.S., Boyer D.S., Kaiser P., Chung C.Y., Kim R.Y. (2006). Ranibizumab for Neovascular Age-Related Macular Degeneration. N. Engl. J. Med..

[47] Brown D.M., Kaiser P., Michels M., Soubrane G., Heier J.S., Kim R.Y., Sy J.P., Schneider S. (2006). Ranibizumab versus Verteporfin for Neovascular Age-Related Macular Degeneration. N. Engl. J. Med..

[48] Suzuki M., Nagai N., Izumi-Nagai K., Shinoda H., Koto T., Uchida A., Mochimaru H., Yuki K., Sasaki M., Tsubota K. (2014). Predictive factors for non-response to intravitreal ranibizumab treatment in age-related macular degeneration. Br. J. Ophthalmol..

[49] Lux A., Llacer H., Heussen F.M.A., Joussen A.M. (2007). Non-responders to bevacizumab (Avastin) therapy of choroidal neovascular lesions. Br. J. Ophthalmol..

[50] Falavarjani K.G., Nguyen Q.D. (2013). Adverse events and complications associated with intravitreal injection of anti-VEGF agents: A review of literature. Eye.

[51] Chen Q.-Y., Chaturvedi P.R., Luesch H. (2018). Process Development and Scale-up Total Synthesis of Largazole, a Potent Class I Histone Deacetylase Inhibitor. Org. Process. Res. Dev..

